# Economic burden of recurrent low back pain among working-age adults in Poland: a 3-year retrospective observational analysis

**DOI:** 10.3389/fpubh.2026.1805628

**Published:** 2026-04-01

**Authors:** Jarosław Szyszka, Jakub Matuska, Bartosz Szyszka, Dariusz Walkowiak, Elżbieta Skorupska

**Affiliations:** 1 Opolskie Center of Rehabilitation, Korfantów, Poland; 2 Department of Physiotherapy, Doctoral School, Poznan University of Medical Sciences, Poznan, Poland; 3 Unit of Histology and Neurobiology, Department of Basic Medical Sciences, Faculty of Medicine and Health Sciences, Rovira I Virgili University, Reus, Spain; 4 Bristol Royal Infirmary, University Hospitals Bristol and Weston NHS Foundation Trust, Bristol, United Kingdom; 5 Department of Organisation and Management in Health Care, Poznan University of Medical Sciences, Poznań, Poland; 6 Department of Physiotherapy, Poznan University of Medical Sciences, Poznań, Poland

**Keywords:** cost analysis, healthcare policy, medical services, recurrent low back pain, working-age

## Abstract

**Introduction:**

Health economic analysis is one of the solutions to optimize healthcare system management. Low back pain (LBP) appears to be increasing in both incidence and financial burden. According to the new pain definition, pain that recurs for more than 3 months should be classified as chronic. However, evidence on the prevalence and economic impact of recurrent LBP (rLBP) in the working-age population remains limited. The aim of this study was to estimate the approximate direct costs of the working-age population with rLBP. Additionally, analyze the cost-per-visit across different age groups and types of medical services.

**Methods:**

A bottom-up framework was used to estimate the national burden of rLBP in Poland, based on data from the Polish National Health Fund and representative hospital records from 2019 to 2021. The analysis included individuals aged 21–65 years with LBP-related ICD-10 diagnoses. Retrospective observational study.

**Results:**

The mean annual direct cost of working-age LBP individuals in Poland was €184,986,519 including €13,699,529 from rLBP (95% CI: €9,829,766–17,522,323). 24.51% of LBP cases were classified as rLBP, covering 54.28% of medical services and 38.37% total LBP costs. The median cost-per-visit for rLBP was significantly higher compared to non-rLBP overall and specific age subgroups (*p* < 0.001). rLBP accounted for 34.72% of total LBP costs in individuals aged over 35 years (over 50 years 23.65%), including 21.56% (over 50 years 14.43%) for inpatient admissions and 10.79% (over 50 years 7.69%) for physiotherapy.

**Conclusion:**

Using the bottom-up framework, the estimated cost of rLBP in Poland was €13,699,529, representing 24.51% of cases in representative hospital data. rLBP showed higher per visit costs than overall LBP across all age groups. Patients over 35 years old accounted for 34.72% of total LBP expenses and two-fifths of all medical services. Moreover, outpatient visits to physiotherapy services were predominant.

## 1 Introduction

Health economic analysis supports evidence-based decision-making aimed at improving healthcare system management. The global problem of ‘work-limiting’ long-term health conditions is continually growing (1, 2). Economic evaluations of cost-effectiveness and cost-efficiency help guide the rational selection of interventions that provide the greatest value. Through economic analyses and health technology assessments, resources can be allocated more efficiently, enabling the design of more sustainable and accessible models of care, including those built on a strong foundation of primary care (3). The World Health Organization (WHO) has developed tools and frameworks to analyze the economic consequences of health outcomes and to support evidence-based decision-making (4). These initiatives have contributed to the increasing role of economic evaluation in healthcare organizations (5, 6).

Getzen et al. argue that as aggregated cost data increase (for example, at the population level), the weaker the relationship between expenditure and actual health outcomes becomes (7). Consequently, analyses conducted at the individual level, such as cost-per-visit assessments, more accurately capture the true variability of service costs (7). Although the per-visit approach is not the dominant standard, it is considered highly useful when evaluating the efficiency of a specific service, identifying inefficiencies, or determining unit costs for use in more advanced economic models (8–11).

This approach is particularly relevant in light of the eleventh revision of the International Classification of Diseases (ICD-11), which expanded the list of codes and conditions (12). ICD-11 was designed to be more accessible and flexible, with simpler coding, digital tools, and concepts tailored for primary care, potentially facilitating adoption among a wide range of healthcare professionals (13, 14). A pertinent example of the value of economic analysis in the context of implementing ICD-11 within national health systems is the newly introduced entry “Chronic Pain” (MG.30). Its adoption requires separate diagnostic and therapeutic pathways, each with its own ICD-11 code, for a condition that was previously diagnosed with a single code. In line with the European Pain Federation (EFIC) recommendations, this approach can support health service delivery by multiple professionals, including physicians, nurses, psychologists, and physiotherapists (15), thus enhancing treatment efficiency. Ultimately, this is expected to lower the overall costs associated with the management of chronic pain, a major global health issue.

At the same time, by categorizing chronic pain as a new disease, attention was directed toward the recurrent state (16). It reclassified a series of pain episodes as chronic pain rather than separate acute or subacute events. At present, there are no robust clinical criteria for defining recurrent pain states (17).

Some chronic diseases, such as low back pain (LBP), appear to be on the rise, both in terms of the occurrence and economic impact (18–21). Evidence indicates that among working-age individuals, LBP can lead to decreased productivity (22–24), increased absenteeism (25–27), and premature retirement (28). These issues are particularly notable among people aged 35–55 years (19, 29–31) and women (11, 32, 33). Alarmingly, the prevalence of chronic low back pain (cLBP) has doubled over the past decade (34). Thus, we can expect an increasing burden on national healthcare systems and the economy due to LBP (30, 35–37), including its chronic state (38–41). According to the European Union Survey on the Living Conditions of the Population, work incapacity affects 20–60% of Poland’s working-age population, depending on age (42). EUROSTAT data further indicate that nearly one in four working adults in Poland suffers from at least two chronic diseases, with cLBP being one of them (1).

Based on the new IASP definition of chronic pain (16), our recent study offered the first accurate estimate of the direct medical costs of recurrent LBP (rLBP) using a cost-per-visit approach. This confirmed the higher costs of rLBP and revealed cost differences associated with sex and the type of health service utilized. Additionally, modelling the annual costs for Poland (approximately €243.9 million per year) emphasized the substantial economic burden of rLBP. This information may support more efficient resource distribution and evidence-based health policy planning (11).

There is limited evidence in the current literature on the prevalence of rLBP (3) and its associated economic burden (33, 43). A study from Japan estimated the annual total medical costs for patients with cLBP at €12,551 (43). Meanwhile, a 2023 meta-analysis indicated that, in high-income countries, the average annual direct costs associated with LBP amount to approximately $9,000 per patient, posing a significant burden on healthcare systems and national economies (37). In light of ICD-11-based chronic pain management (44), and the high prevalence of LBP chronicity with age, it is vital to determine the cost differences based on age ranges (3, 5, 21) and the medical services provided by multidisciplinary teams (45–48).

In this study, rLBP is approached as a specific pattern of healthcare utilization associated with chronic pain, rather than as a diagnosis defined exclusively by symptom duration. By focusing on repeated episodes that require ongoing medical attention, this approach enables the identification of cost drivers related to service type and patient age, which may remain obscured in aggregated cost analyses. In the context of the ICD-11 classification of chronic pain, such an economic perspective aligns with the emphasis on care pathways and multidisciplinary management, offering insights relevant to the optimization of healthcare resource allocation. Therefore, based on a three-year retrospective observational study, we aimed to estimate the costs incurred by the working-age population with rLBP. Additionally, we examined differences in cost-per-visit across age groups and types of medical services.

## 2 Materials and methods

The retrospective observational investigation was conducted between December 2023 and February 2024. According to Polish legal regulations and the General Data Protection Regulation, ethics committee approval was not required because the dataset was fully anonymized and contained no information allowing the identification of individuals. Furthermore, we obtained an official statement confirming that the project does not qualify as an experiment and is therefore not subject to the jurisdiction of the Bioethics Committee at the Poznan University of Medical Sciences (KB-496/23). Based on this determination, patient consent was deemed unnecessary, as the study did not involve experimental procedures or the processing of personal data. This study was reported in accordance with the Strengthening the Reporting of Observational Studies in Epidemiology (STROBE) guidelines (49).

### 2.1 Data sources

Data on the volume of medical services and total treatment expenditures for working-age patients with LBP in Poland were obtained from the Polish National Health Fund (NHF) for the years 2019–2021. The dataset was classified using ICD-10 codes G54, G55, M45, M46, M47, M48, M49, M51, M53, and M54, and included patients’ age and the monetary value of services associated with these diagnoses. The data were further stratified by voivodeship and county, as well as by type of healthcare delivery, encompassing primary care, outpatient specialist services, and inpatient hospital care. The Opolskie Centre of Rehabilitation (OCR) was selected as the reference provider because of its comprehensive and standardized service structure, enabling accurate estimation of unit costs and cost-per-visit values across different types of care. The study population consisted of working-age adults (21–65 years) with LBP-related ICD-10 diagnoses corresponding to the codes listed above. Individuals with malignancies, traumatic injuries, or chronic inflammatory disorders were excluded from the analysis.

### 2.2 Study sample and data analysis

We assessed the total costs of LBP-related medical services provided to working-age adults at OCR during the 2019–2021 observation period, categorized by ICD-10 codes and recurrent status, as outlined in the new chronic pain definition (17, 50). Regarding the definition of rLBP (51), it underscores the difficulty of adopting a single, uniform definition for this condition (50). However, based on the work of de Vet (52), rLBP can be defined as episodes of LBP occurring after periods without symptoms or after periods during which the patient has not sought medical care for LBP (53). De Vet also recommends a minimum 30-day interval between symptom resolution and recurrence. Since chronic pain is defined as lasting longer than 12 weeks and patients may not seek care immediately after symptoms resolve, a 30-day pain-free interval was adopted as the operational threshold (52). Although a 30-day pain-free interval is commonly used in clinical definitions of recurrent LBP, applying this threshold in administrative healthcare datasets is challenging, as symptom resolution and pain-free periods are not directly observable. Therefore, to minimize the risk of misclassifying ongoing treatment episodes as true recurrences and to better reflect real-world healthcare utilization patterns, a six-month interval between recorded medical encounters under the same ICD-10 code was adopted as the operational definition of recurrence. Recognizing that patients frequently postpone seeking medical attention after symptom recurrence, and aiming to align with real-world healthcare utilization patterns, we defined rLBP in our dataset as any subsequent medical service recorded under the same ICD-10 code occurring at least 6 months after the initial encounter. This operational definition was intended to capture clinically meaningful recurrence as reflected in routine medical records. Based on this criterion, patients were subsequently categorized according to the number of revisits occurring no earlier than 6 months after the original LBP diagnosis. Individuals were assigned to either the non-recurrent or recurrent LBP group, with recurrent LBP defined as having three or more return visits for the same diagnostic category.

Additionally, analyses were stratified by age group—G1 (21–35 years), G2 (36–50 years), and G3 (51–65 years)—and by type of medical service received, including inpatient admissions (orthopedics and rehabilitation wards), outpatient consultations, and physiotherapy services. It should be noted that patients could use more than one type of service (inpatient, outpatient, or physiotherapy). When reporting the distribution of service types utilized by patients, the total percentage may exceed 100%.

**Figure 1:**
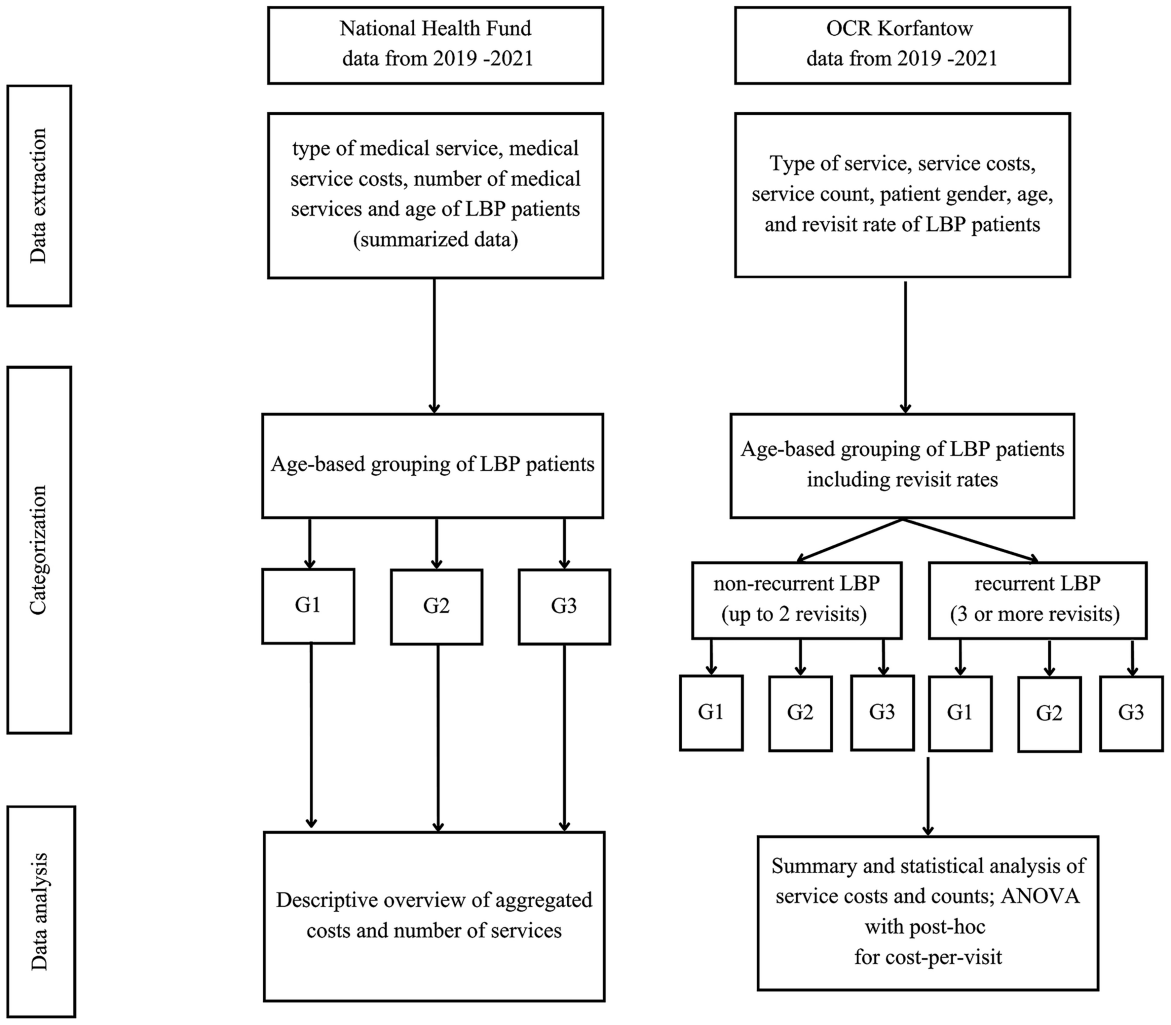
Flow diagram of the study design.

Excluding patients with mixed or non-recurrent patterns may overlook individuals who experience intermittent or infrequent episodes of LBP, potentially underestimating the overall burden of low back pain across diverse patient groups. This limitation may also reduce the generalizability of the findings to the broader LBP population. Moreover, applying a 6-month interval between medical encounters may omit patients who seek care within shorter time frames, and the interchangeable use of various ICD-10 codes for similar clinical presentations may result in incomplete case identification. Nonetheless, the exclusion criteria were implemented to create a more homogeneous cohort for the analysis of recurrent LBP, and we consider the resulting bias minimal given the study design. The full process of data extraction, classification, and analysis is illustrated in Figure 1.

We observed a comparable age distribution (11:34:55, G1:G2:G3) in the percentage share of service value across Poland, Opole Voivodeship, and OCR.

A prevalence-based bottom-up framework was applied to estimate the national burden of rLBP in Poland (54–56). The analysis was informed by OCR records, which provided age-specific frequencies of medical services delivered to patients with rLBP. These frequencies were used to project the expected number of corresponding services performed nationally between 2019 and 2021. To assign monetary values to individual procedures, NHF reimbursement tariffs were applied to each OCR service. The mean cost per service, along with its 95% confidence interval (95% CI), was estimated using a bootstrap method in which 5,000 resamples were generated, with each service having a 50% probability of selection in each iteration.

The projected national cost of rLBP was calculated by multiplying the estimated number of services in Poland by the mean OCR service value. This method provided an age-stratified estimate of the overall financial burden attributable to rLBP. To derive the annual cost, the three-year cumulative estimate was divided by three. During the study period, inflation remained low, the exchange rate was stable, and contractual settlement rules for these services did not materially change. Accordingly, values expressed in euros (€) were calculated using the original prices in PLN and an exchange rate of 1€ = 4.35 PLN (National Bank of Poland, November 27, 2023).

### 2.3 Statistical analysis

The data analysis involved several methodological steps. First, the cost-per-visit was examined. The Shapiro–Wilk test assessed the data’s distribution and verified assumptions about normality. Due to the nonparametric nature of the dataset, descriptive statistics—including the median and interquartile range—were used to summarize central tendencies and variability. To compare costs across age groups, the Mann–Whitney U test was employed, as it provides a robust nonparametric alternative for assessing differences in distributions. Additionally, the Kruskal–Wallis ANOVA evaluated cost variation across multiple patient subgroups. *Post hoc* comparisons were conducted using the Dunn–Benjamini–Hochberg procedure, which adjusts for multiple testing while controlling the false discovery rate and maintaining a suitable balance between Type I and Type II error risks, providing greater sensitivity than more conservative corrections such as Bonferroni. In a further analytical step, mean costs were compared stratified by age group and type of medical service. Homogeneity of variance was assessed using Levene’s test, and Welch’s t-test was applied to evaluate differences in mean costs, given its suitability for datasets with unequal variances.

## 3 Results

According to NHF records from 2019 to 2021, the mean annual direct expenditure on medical care for working-age individuals with LBP in Poland was €184,986,519, with an average of 218,214 services delivered annually. When stratified by age, the mean yearly number of services was 53,942 in G1, 79,052 in G2, and 85,220 in G3.

### 3.1 The simulated age-groups cost estimates for recurrent LBP in Poland for the years 2019–2021

An analysis of the OCR dataset from 2019 to 2021 allowed us to derive age-group-specific estimates of the unit cost for rLBP–related procedures, which were subsequently extrapolated to national figures. When these group-level cost estimates were applied to the corresponding number of procedures performed in Poland, the combined annual financial burden attributable to rLBP was calculated at €13,699,529, with a 95% confidence interval of €9,829,766 to €17,522,323. Each age group contributed differently to the overall expenditure. For G1, the annual cost associated with recurrent LBP was estimated at €2,900,163 (95% CI: € 1,569,901 - €4,216,081). In G2, the projected annual expenditure was €4,499,310, with an uncertainty interval from €3,233,881 to €5,763,650. The highest cost was observed in G3, with an annual estimate of €6,300,056 (95% CI: €5,025,984 - €7,542,592).

### 3.2 Three-year retrospective data from a working-age LBP cohort hospitalized at OCR Korfantow

At OCR, the analyzed cohort of working-age LBP patients (n = 2,126) received a total of 5,098 medical interventions, generating cumulative costs of €857,295. The mean annual direct cost of LBP-related services was €285,765, corresponding to 1,699 procedures.

In the working-age LBP cohort, we observed a statistically significant exponential increase in the proportion of patients with advancing age (*p* < 0.001), with age-group distributions of 11.85% in G1, 34.25% in G2, and 53.9% in G3 (*p* < 0.000001). A similar pattern was evident in the utilization of medical services: the total number of services increased progressively across age groups, with G1 accounting for 11.07%, G2 for 34.44%, and G3 for 54.49% of all services. Throughout the entire LBP population, the number of medical encounters, total expenditures, and cost-per-visit increased with age, with patients aged 50 years and older (G3) exhibiting the highest resource use (Table 1).

**Table 1:** Data of LBP patients in OCR overall and across age groups.

Group	Sum of costs € (%)	Cost-per-visit M, IQR
Total	857,295	98 18–387
G1	86,761 (10.12)	31 31–268
G2	293,573 (34.25)	88^a^ 18–313
G3	476,961 (55.63)	273^b,c^ 20–530
Inpatient admission	561,468 (65.54)	550^1*, 2*^ 550–1,211
Outpatient admission	293,573 (4.46)	18 9–34
Physiotherapy	257,568 (30.00)	275 ^3*^ 131–303

Percentages represent the proportion of the total number of medical services and the proportion of total direct LBP-related healthcare costs within the OCR cohort, stratified by age group and type of medical service. Cost per visit is presented as median (M) with interquartile range (IQR). Non-rLBP refers to low back pain cases without recurrent episodes, whereas rLBP denotes recurrent low back pain. G1: 21–35 years; G2: 36–50 years; G3: 51–65 years. Statistical comparisons between age groups were performed using the Kruskal–Wallis test, with *post hoc* pairwise comparisons indicated as follows: a—G1 vs. G2; b—G1 vs. G3; c—G2 vs. G3. Comparisons between types of medical services were conducted using the Mann–Whitney *U* test and are denoted as follows: 1*—inpatient vs. outpatient care; 2*—inpatient vs. physiotherapy; 3*—physiotherapy vs. outpatient care.

The distribution of specific medical services also varied by age. Among all LBP cases, inpatient admissions accounted for 7.88% of services in G1, 30.98% in G2, and 61.14% in G3. Physiotherapy services represented 9.79, 31.23, and 58.98% across the same groups, whereas outpatient consultations accounted for 14.96, 36.48, and 48.56%, respectively. The highest median cost-per-visit in the OCR cohort was associated with inpatient admissions. A detailed summary of median per-visit costs, total costs, and the number of services across age groups is presented in Table 1.

### 3.3 Recurrent LBP cohort analysis

The rLBP group represented 24.51% of all working-age LBP patients, 54.28% of all LBP medical services, and 38.37% of total LBP costs. The proportion of patients in the rLBP group increased with age, accounting for 2.35% in G1, 7.86% in G2, and 14.30% in G3, and was significantly lower (*p* < 0.001) compared to the non-rLBP group, which had shares of 9.50% in G1, 26.38% in G2, and 39.61% in G3. Furthermore, both groups showed a substantial increase in the number of patients with increasing age (*p* < 0.001). A similar trend was observed in the number of medical services, with the rLBP group accounting for 4.79% in G1, 18.01% in G2, and 23.40% in G3, whereas the non-rLBP group accounted for 6.28% in G1, 16.43% in G2, and 32.40% in G3.

The analysis revealed that the median cost-per-visit for rLBP was significantly higher compared to non-rLBP (*p* < 0.001) overall and in specific age subgroups (*p* < 0.001). In rLBP, the cost-per-visit remained high across all age groups, whereas in non-rLBP it increased gradually with age, aligning with the overall LBP trend. The G1 subgroup, although small, had an unexpectedly high cost-per-visit but accounted for only 3.66% of total rLBP costs. Despite constituting a minority of the overall working-age LBP population, patients with recurrent LBP accounted for a disproportionately large share of healthcare utilization and direct medical costs. Notably, in rLBP, patients aged 35 and older (G2, G3) accounted for 22.16% of all LBP cases, 34.72% of costs, and 41.41% of services. Detailed data is presented in Table 2.

**Table 2:** Data of LBP patients subdivided into recurrent (rLBP) and non-recurrent (non-rLBP) groups overall and across age groups.

	Group	Non-rLBP		rLBP		*P*-value UMW Cost-per-visit
		Sum of costs € (%)	Cost-per-visit M, IQR	Sum of costs € (%)	Cost-per-visit M, IQR	
	Total	528,282 (61.62)	98 18–387	329,013 (38,37)	199 73–535	<0.001
Age	G1	55,399 (6.46)	20 12–140	31,362 (3.66)	241 77–454	<0.001
	G2	198,694 (23.18)	45^a^ 17–303	94,879 (11.07)	173 70–409	<0.001
	G3	274,189 (31.97)	263^b, c^ 20–498	202,772 (23.65)	294 81–598	<0.001

Percentages represent the proportion of total direct LBP-related costs within the recurrent (rLBP) and non-recurrent (non-rLBP) subgroups, both overall and across age groups; Cost per visit is presented as median (M) with interquartile range (IQR). Non-rLBP refers to low back pain cases without recurrent episodes, whereas rLBP denotes recurrent low back pain. G1: 21–35 years; G2: 36–50 years; G3: 51–65 years. Statistical comparisons between age groups, within rLBP and non-rLBP groups, were performed using the Kruskal–Wallis test, with post hoc pairwise comparisons indicated as follows: a—G1 vs. G2; b—G1 vs. G3; c—G2 vs. G3. Comparisons between rLBP and non-rLBP services were conducted using the Mann–Whitney *U* (UMW).

### 3.4 The cost analysis depended on age, type of medical services, and rLBP

The analysis of the distribution of medical services by patients’ recurrence status showed that, in inpatient admissions, the rate of rLBP was 3.71%, compared with 13.59% for non-rLBP. In outpatient care, it was 23.04% for rLBP versus 42.34% for non-rLBP. For physiotherapy services, the rates were 15.71% for rLBP and 27.51% for non-rLBP. A detailed data analysis of rLBP versus non-rLBP by age subgroups was as follows: G1: inpatients 0.47% vs. 0.89%, outpatients 2.30% vs. 7.48%, physiotherapy services 1.60% vs. 2.63%; G2: inpatients 1.08% vs. 4.28%, outpatients 7.43% vs. 16.42%, physiotherapy services 4.94% vs. 8.56%; and G3: inpatients 2.16% vs. 8.42%, outpatients 13.31% vs. 18.44%, physiotherapy services 9.17% vs. 16.32%.

Patients aged 35 years or older (G2 + G3) in the non-rLBP subgroup accounted for 56.15% of total costs across the LBP population, including 36.52% for inpatient admissions and 19.63% for physiotherapy. The second-highest cost was incurred by patients aged 35 years and older in the rLBP subgroup, accounting for 34.72% of all costs, including 21.56% for inpatient admissions and 10.78% for physiotherapy (Table 3). The analysis of cost-per-visit indicates a statistically significant higher treatment cost for rLBP compared to non-rLBP in outpatient admissions across all age groups, as well as in inpatient admissions for patients over 35 years of age (G2 and G3). Furthermore, an exponential increase in physiotherapy costs with age was observed in both the rLBP and non-rLBP groups. Additionally, an age-related increase in total treatment costs was observed only in the rLBP group, whereas inpatient treatment costs decreased with age among patients with rLBP. Detailed data, divided by age groups, is presented in Table 3.

**Table 3:** Data of low back pain (LBP) patients subdivided into recurrent (rLBP) and non-recurrent (non-rLBP) groups overall and across age groups with division on type of services.

Type of service	Group	Sum of costs € (%)			Cost-per-visit M, IQR		
		G1	G2	G3	G1	G2	G3
Inpatient Admission	rLBP	22,115 (2.58)	61,101 (7.13)	123,699 (14.43)	2,250 774–2,654	2,654 1,169–3,942	1885 550–4,050
	non-rLBP	41,452 (4.84)	147,964 (17.26)	165,137 (19.26)	2654^ab^ 1,049–3,344	922^c^ 550–2,654	550 550–881
*P*–value UMW		–	–	–	0.517	0.001	<0.001
Outpatient Admission	rLBP	1,586 (0.18)	7,283 (0.85)	13,168 (1.54)	25 18–36	36^a^ 21–59	36^b^ 21–59
	non-rLBP	2,895 (0.33)	6,658 (0.78)	6,669 (0.78)	17 8–18	17 9–24	16 9–20
*P*–value UMW		–	–	–	<0.001	<0.001	<0.001
Physiotherapy	rLBP	7,661 (0.89)	26,495 (3.09)	65,905 (7.69)	157 66–330	194 116–333	283^bc^ 125–467
	non-rLBP	11,052 (1.29)	44,073 (5.14)	102,382 (11.94)	175 68–303	264^a^ 118–303	283^bc^ 257–303
*P*-value UMW		-	-	-	0.546	0.956	0.659

Percentages represent the proportion of the total low back pain (LBP) patient population, stratified into recurrent (rLBP) and non-recurrent (non-rLBP) subgroups, both overall and across age groups. Age groups were defined as G1, 21–35 years; G2, 36–50 years; and G3, 51–65 years. Cost data are presented as median (M) with interquartile range (IQR). Between-group comparisons across age groups were performed using the Kruskal–Wallis test, with *post hoc* pairwise comparisons indicated as follows: a, G1 vs. G2; b, G1 vs. G3; and c, G2 vs. G3. Comparisons between rLBP and non-rLBP groups were performed using the Mann–Whitney *U* test (*p < 0.05).

## 4 Discussion

To the best of our knowledge, this study represents the first attempt to comprehensively quantify the direct healthcare costs associated with rLBP in the working-age population. Notably, the cost analysis was primarily based on cost-per-visit as the principal metric. We also examined the data by grouping it according to age and type of medical service as parameters for the cost analysis. The annual direct cost attributable to rLBP was estimated at €13,699,529, representing approximately 7.4% of the overall direct expenditures for LBP. Our findings indicated that the highest treatment costs among working-age individuals with LBP were observed in those over 50 years old. Care for this group accounted for more than half of total direct costs and involved more than 50% of all healthcare services provided. Patients with rLBP comprised nearly a quarter of the overall LBP population. Within this subgroup, individuals aged 50 and older were responsible for 23.65% of all direct LBP-related costs and received one-fourth of all LBP-related healthcare services. The substantial cost of treating rLBP, regardless of age, is further supported by the per-visit analysis, which highlights the significant role of inpatient and physiotherapy care in driving overall costs (Tables 2, 3).

There is no universally accepted definition in the literature for a recurrent state of chronic pain syndrome (16, 50). One proposed definition describes rLBP as the occurrence of three or more consecutive visits for the same clinical presentation, initiated no earlier than 6 months after the initial diagnosis and occurring within a three-year observation period (11). To date, our investigation remains one of only two studies to evaluate the economic consequences of rLB (11, 57). Based on our two studies, we demonstrated that the proportion of individuals with rLBP is higher in working-age groups than in the overall LBP population (11). Earlier studies have reported recurrence rates ranging from 24 to 80%, but these data mainly reflected chronic pain syndrome, specifically interrupted LBP symptoms within a one-year period (58–61). As noted previously, the absence of standardized clinical criteria for defining recurrence limits comparability across studies and may contribute to the substantial heterogeneity in reported recurrence rates (17, 50, 51, 62).

We found that one-quarter of all working-age individuals with LBP were classified as having recurrent episodes. Notably, this subgroup accounted for nearly 40% of total direct healthcare costs, driven primarily by their use of more than half of the provided health services, particularly inpatient care and physiotherapy. There is limited data on the overall direct costs of cLBP. It has been reported that in Sweden, it accounted for 15% of the total direct cost of LBP (63), whereas in South Africa, it accounted for up to 83% (64). Interestingly, Mehra et al. reported that patients with cLBP with a neuropathic component generated almost all (96%) of the direct medical costs of LBP, even though they constituted only a portion of the patient population (65). This is particularly relevant considering ICD-11’s requirements for the management of chronic pain syndrome, in which specific codes depend on the underlying pain mechanism, such as pain perpetuation (e.g., neuropathic pain).

Both the size of the working-age rLBP population and the direct costs associated with their management clearly highlight the need to treat this subgroup as a distinct clinical category. This issue becomes even more relevant with the implementation of the MG.30 category within ICD-11 (12, 13), as the introduction of this new diagnostic unit necessitates system-level adjustments to healthcare service allocation. In line with WHO recommendations, the management of conditions classified as chronic primary pain (MG30.0) is expected to rely more heavily on physiotherapy, often combined with cognitive-behavioral therapy (66, 67). The cost-per-visit framework proposed in our study — an approach seldom applied in previous health-economic analyses — serves as a valuable tool for evaluating treatment expenditures and guiding the restructuring of service delivery in line with ICD-11 recommendations (41). Prior research has demonstrated considerable variability in the distribution of direct healthcare costs among LBP populations, with recurrent or high-utilization subgroups accounting for a disproportionate share of expenditures (55, 68).

In our analysis, we found that patients with rLBP incurred significantly higher per-visit costs than those without rLBP, and these elevated costs remained consistently high across all age groups (Table 2). Similarly, for cLBP, substantially higher unit costs per patient and per visit were observed compared to acute or non-specific LBP (40, 69). The difference was especially evident in subgroups with a neuropathic component (65). A more detailed review of age-related service utilization patterns further suggests that the traditional focus on individuals aged 50 and older may be insufficient for rLBP, as increased costs were evident from age 35 onward. Notably, patients aged 35 and older in the rLBP subgroup accounted for nearly one-third of total expenditures, including approximately one-fifth attributable to inpatient admissions and just over 10% to physiotherapy (Table 3). Chronic LBP is associated with significantly higher hospital treatment costs than acute LBP. Limiting the analysis to those over 50 increases this disparity, as older patients are more likely to experience chronic pain, requiring more intensive and costly inpatient stays and specialist procedures. Other researchers have also confirmed that direct expenses vary significantly depending on the type of service, with hospital admissions incurring higher costs than outpatient care or physiotherapy services (70, 71). Therefore, despite the low proportion of cLBP patients using this type of treatment, optimizing it in line with the latest advances in pain medicine will be a key step in reducing future direct costs.

Accordingly, our findings support prior literature indicating that a per-visit analytical approach facilitates the identification of medical service types that are cost-intensive or potentially inefficient. Cost-per-visit constitutes a key parameter for improving the efficiency of healthcare resource allocation across different service areas. We propose that the median cost-per-visit provides a more stable and informative measure of the relationships between cost and the factors examined. This metric is particularly relevant in chronic pain management, where the biopsychosocial framework underscores the importance of coordinated, multidisciplinary interventions that collectively improve therapeutic outcomes (44, 45, 72).

In the context of chronic pain (ICD-11: MG.30), there is a growing emphasis on reorganizing health service management by structuring care for cLBP around four key clinical professions: physicians, nurses, physical therapists, and psychologists (73–75). At the same time, there is widespread emphasis on aligning diagnostic and therapeutic practices with the latest WHO recommendations, which highlight the importance of a biopsychosocial approach to the treatment of chronic pain (76, 77). Furthermore, long-standing scientific evidence indicating that routine imaging should not be a standard diagnostic procedure for chronic musculoskeletal disorders, including nonspecific low back pain, strengthens the case for evidence-based services (78, 79). In summary, these findings support the view that combining clinical guidelines with economic analyses provides a solid foundation for rational and sustainable healthcare system reforms (80, 81).

In the absence of effective therapeutic options, patients with rLBP often make repeated attempts to seek care, including additional diagnostic evaluations and pharmacological interventions. Our study confirmed that only 15.71% of these patients use physiotherapy. Importantly, this low utilization occurs despite physiotherapy contributing to higher per-visit cost estimates, indicating that its role in cost generation reflects higher unit prices rather than overuse. The relatively small proportion of physiotherapy services within the rLBP group suggests a potential mismatch between current service usage patterns and evidence-based guidelines for chronic pain management. This raises the question of whether the high volume of services provided to patients with rLBP in outpatient settings, accounting for approximately 23% of all visits for LBP, and the higher cost-per-visit compared to patients with non-rLBP, should be partly shifted towards primary care, especially toward physiotherapy. Other authors have estimated that physiotherapy accounts for 20–32% of direct healthcare costs to date (82). Furthermore, despite increasing total expenditure per patient, a series of physiotherapy visits significantly improves treatment effectiveness, reducing costs by limiting the need for more expensive services, such as hospital admission or pharmacological treatment (82). Similar concerns regarding inefficient allocation of resources in LBP pathways have been raised in recent health services research (81, 83).

It can further be assumed that patients reporting recurrent pain symptoms, experienced for example over 2 weeks, are commonly referred by primary care physicians for additional specialist diagnostics. However, in light of current evidence and the classification of rLBP as a form of chronic pain, such referral patterns seem inconsistent with contemporary clinical recommendations (76, 77, 83). Current models of chronic pain management emphasize differentiating diagnostic and therapeutic approaches according to the predominant phenotype perpetuating pain, with clinical assessment primarily guided by physical examination and standardized self-report questionnaires (16). Conversely, routine use of imaging, such as MRI or ultrasound, or laboratory tests is recommended only when a differential diagnosis is required, or red-flag symptoms are present (84, 85).

From an economic perspective, future research should incorporate a more accurate analysis of the rLBP inpatient group. The expense of treating this subgroup represents nearly a quarter (25%) of all LBP costs, with almost 15% attributable to patients aged 50 years and older. This prompts inquiry into the potential influence of this particular group on the development of the phenomenon known as post-surgical low back pain syndrome (PSLBP), also known as failed back surgery syndrome (FBSS), which affects up to 40% of patients (86, 87).

It is plausible that, in a subset of patients, persistent pain results from central sensitization processes that do not respond to interventional treatments, necessitating multimodal therapy that excludes invasive methods. The literature suggests that up to 30% of LBP cases may exhibit features of central sensitization in pain persistence (88). The treatment approach for chronic pain recommended by the WHO and EFIC, which acknowledges the vital role of physiotherapy combined with cognitive-behavioral therapy (CBT) (47, 77), should perhaps precede surgical procedures, especially in the rLBP group aged 50 and over (Table 3).

From a clinical perspective, 90% of LBP cases are classified as non-specific pain, meaning that in chronic and especially recurrent states, this remains the predominant patient group. For this group, the WHO does not recommend pharmacotherapy beyond NSAIDs, but instead highlights the important role of modern physiotherapy in collaboration with a psychologist specializing in chronic pain (76, 89). Consequently, implementing these recommendations in national healthcare systems is hypothesized not only to improve treatment efficacy but also to significantly reduce treatment costs, as corroborated by our analyses using per-visit metrics and overall rLBP expenditure. A key finding of our study, in the context of the proposed changes to ICD-11, is the disproportionately low utilization of physiotherapy services among the working-age LBP population.

The overall usefulness of the results is based on identifying significant costs of rLBP treatment and analyzing per-visit costs, considering the type of medical service. These data appear to have a utilitarian nature, which should be considered. Current medical recommendations for MG. Thirty favor allocating outpatient services to outpatient physiotherapy, particularly for patients with chronic primary LBP. However, the results of the cost simulation for the whole country should be approached with caution.

Given the previously discussed findings, it is essential to pursue further research encompassing the entire population with cLBP, with particular emphasis on individuals aged 50 and older. Analyses of both total healthcare expenditures and per-visit costs consistently demonstrate that the highest treatment expenses are incurred in this age cohort. This segment represents more than half of the total LBP population, accounts for approximately half of all treatment costs, and utilizes roughly half of all medical services and benefits.

### 4.1 Future perspective

Considering the substantial financial burden associated with rLBP, prioritizing interventions specifically designed for this subgroup could improve clinical outcomes and reduce overall healthcare expenditure. Consequently, future research should explore the long-term impacts and cost-effectiveness of different treatment modalities for cLBP, including analyses at the per-visit level. A detailed examination of specific non-invasive treatment components—such as manual therapy and structured exercise programs—may offer additional insights. Moreover, in some instances of chronic pain, neuromodulation may represent a more cost-effective alternative to traditional treatment pathways.

### 4.2 Limitations

Several factors limit the findings of this study. Primarily, there is a deficiency of data pertaining to the costs associated with primary healthcare and consultations. Additionally, data from departments favoring surgical procedures are missing. Furthermore, the analysis of inpatient admissions should differentiate between invasive and non-invasive therapeutic procedures. The selection of the study population necessitates precise diagnostic coding for each diagnosis associated with specific treatments or provider visits. Also, due to the retrospective nature of the study, limitations include the absence of data on comorbidities, income levels, healthcare access, work disability, and related indirect costs.

Furthermore, in Poland, healthcare services are primarily financed by the National Health Fund. Future research should consider variables such as national healthcare policies, income levels, and access to private healthcare. The period during which the study was conducted coincided with the COVID-19 pandemic, which may have affected the results. Since this study does not analyze epidemiological data but instead explores the impact of rLBP on economic assessments, it is advisable for future studies to cover a 10-year period to facilitate additional observations. Additionally, a multicenter approach would likely yield more precise economic evaluations and robust bottom-up cost estimates.

## 5 Conclusion

Using the bottom-up framework, the estimated cost of rLBP in Poland was €13,699,529, representing 24.51% of cases in representative hospital data. rLBP showed higher per-visit costs than overall LBP across all age groups. Patients over 35 years old accounted for 34.72% of total LBP expenses and two-fifths of all medical services. Moreover, outpatient visits to physiotherapy services were predominant. Future health economic research should prioritize the assessment of the justification for surgical interventions in cases of rLBP.

## Data Availability

The raw data supporting the conclusions of this article will be made available by the authors, without undue reservation.
